# Is hybrid therapy more efficient in the eradication of *Helicobacter pylori* infection? A systematic review and meta-analysis

**DOI:** 10.1186/s12941-023-00582-2

**Published:** 2023-07-04

**Authors:** Maria José Temido, Dara Mbanze, Nuno Almeida, Bárbara Oliveiros, Elisa Gravito-Soares, Pedro Figueiredo

**Affiliations:** 1 Gastroenterology Department, Centro Hospitalar e Universitário de Coimbra, Praceta Prof. Mota Pinto, 3004-561 Coimbra, Portugal; 2 Faculty of Medicine, University of Coimbra, Coimbra, Portugal; 3 Centro Hospitalar e Universitário de Coimbra, Praceta Prof. Mota Pinto, 3004-561 Coimbra, Portugal

**Keywords:** *Helicobacter pylori*, Hybrid therapy, Sequential therapy, Concomitant therapy, Meta-analysis

## Abstract

**Introduction:**

Hybrid therapy (HT) is a non-bismuth quadruple therapy created to surpass *Helicobacter pylori*’s (*H. pylori*) resistance rates to antibiotics. HT has excellent eradication rates, as well as a very good compliance and safety profile. We aim to compare HT with sequential therapy (ST) and concomitant therapy (CT) for the eradication of *H. pylori*.

**Methods:**

This systematic review was conducted following the principles of the PRISMA guidelines. Literature was electronically searched on the CENTRAL library, PubMed, Embase, Scopus, LILACS, and ClinicalTrials.gov. Only randomized controlled trials were included. The primary outcome evaluated was eradication rate of *H. pylori*. The secondary outcomes evaluated were adverse events and compliance rates. Meta-analyses were performed with Cochrane Review Manager 5.4. The Mantel–Haenszel method was used to estimate the pooled relative risk and 95% confidence interval of the eradication rates between HT and other regimens, as well as the secondary outcomes.

**Results:**

10 studies were included, comprising 2993 patients. The mean eradication rates achieved by HT with intention-to-treat (ITT) and per-protocol (PP) analyses were, respectively, 86% (range: 79.2–90.8%) and 91.7% (range: 82.6–96.1%). No statistically significant difference was found in ITT eradication rate between HT and CT (relative risk: 1; 95% CI: 0.96- 1.03) and between HT and ST (relative risk: 1.02; 95% CI: 0.92–1.14). PP analysis revealed similar results. HT was associated with higher compliance rates than CT and slightly lower than ST. As far as adverse events are concerned, this meta-analysis demonstrated a higher occurrence of adverse events on the group of patients treated with CT when compared with HT. HT and ST showed similar results.

**Conclusion:**

HT has similar eradication, compliance and adverse event rates when compared to ST, but a better safety profile than the CT.

**Supplementary Information:**

The online version contains supplementary material available at 10.1186/s12941-023-00582-2.

## Introduction

*Helicobacter pylori* (*H. pylori*) infection is one of the most prevalent infections worldwide [1]. In fact, *H. pylori* is one of the most important carcinogenic factors contributing to gastric cancer [2]. Recent studies have revealed that *H. pylori* eradication leads to lower rates of this malignancy [3]. Taking all these factors into consideration, optimization of *H. pylori* eradication therapies is of utmost importance [4, 5].

Even though this bacterium’s discovery took place more than 40 years ago [6], there are still major challenges regarding its eradication [7]. The most successful treatment regimen is yet to be determined. The four most used antibiotics are: metronidazole, clarithromycin, amoxicillin, and tetracycline [8]. This fact results from the efficacy of these therapies and relatively low rates of side effects. Nevertheless, there has been a recent rise in bacterial resistance to these drugs: firstly, to metronidazole and later to clarithromycin. As a result, treatment must include more than one antibacterial with different mechanisms of action, to obtain an effective result [6, 9]. The already proposed regimens are: triple therapy (TT) (proton pump inhibitor (PPI) and two antibiotics, clarithromycin and amoxicillin or metronidazole), non-bismuth quadruple therapy (PPI, clarithromycin, metronidazole, and amoxicillin) and bismuth quadruple therapy (PPI, bismuth salt, tetracycline, and metronidazole) [10]. Efficacy of TT has been declining as resistance rates are evolving [11] and previous works have outlined that efficacy of TT is insufficient. [12–14]

Bacterial gene mutations seem to play a major role in the resistance [10]. In many countries, primary clarithromycin and metronidazole resistance rates are higher than 15% and combined resistance rates to clarithromycin and metronidazole are around 10% [15]. In Portugal, the resistance rates are as high as 40–50% to clarithromycin and around 25–30% to metronidazole. [16, 17]

Hybrid therapy (HT) is a quadruple non-bismuth therapy, which functionally is a combination of sequential and concomitant therapies. HT consists of a proton pump inhibitor (PPI) and amoxicillin for 10 to 14 days, adding clarithromycin and metronidazole in the final 5 to 7 days of treatment. The original clinical trial demonstrated an eradication rate of 99.1% (95% confidence interval (CI): 97.3–100.9%) according to per-protocol (PP) analysis and 97.4% (95%CI: 94.5–100.3%) by intention-to-treat (ITT) analysis [18, 19].

In recent years, HT has been gaining attention as a potentially, more successful therapy, showing better results eliminating this bacterium when compared to other treatment regimens in several clinical trials [9]. Nevertheless, the conclusions of the studies were not consensual. Some randomized clinical trials revealed conflicting results, not being concordant on whether HT was better at eradicating *H. pylori* than ST [20–23].

Knowing the most efficacious therapy regimen is imperative since *H. pylori* infection is responsible for losses in health-related quality of life and deaths worldwide. We thus aim to compare the effectiveness of HT in the eradication of *H. pylori* with other recommended therapeutic regimens: sequential and concomitant therapies. Moreover, we aim to compare the adverse events and compliance rates between the above-mentioned therapies.

## Materials and methods

### Protocol and registration

This systematic review and meta-analysis was conducted according to the Preferred Reporting Items for Systematic Reviews and Meta-analyses (PRISMA) Statement [24].

The protocol of the present review was registered in the PROSPERO (International Prospective Register of Systematic Reviews) database under the identification number CRD42022314599.

### Eligibility criteria

To define our eligibility criteria, we referred to the PICO (Population; Intervention; Comparison; Outcome) framework, according to the current PRISMA guidelines.

Our population was defined as adults (older than 18 years-old) with confirmed infection by *H. pylori*, with or without dyspeptic symptoms. *H. pylori* infection diagnostic methods were defined as follows: endoscopy with biopsies of the stomach, with either histological examination, gram staining or rapid urease test; urea breath test and/or stool antigen test. Only studies addressing treatments as first-lines were included.

**Fig. 1:**
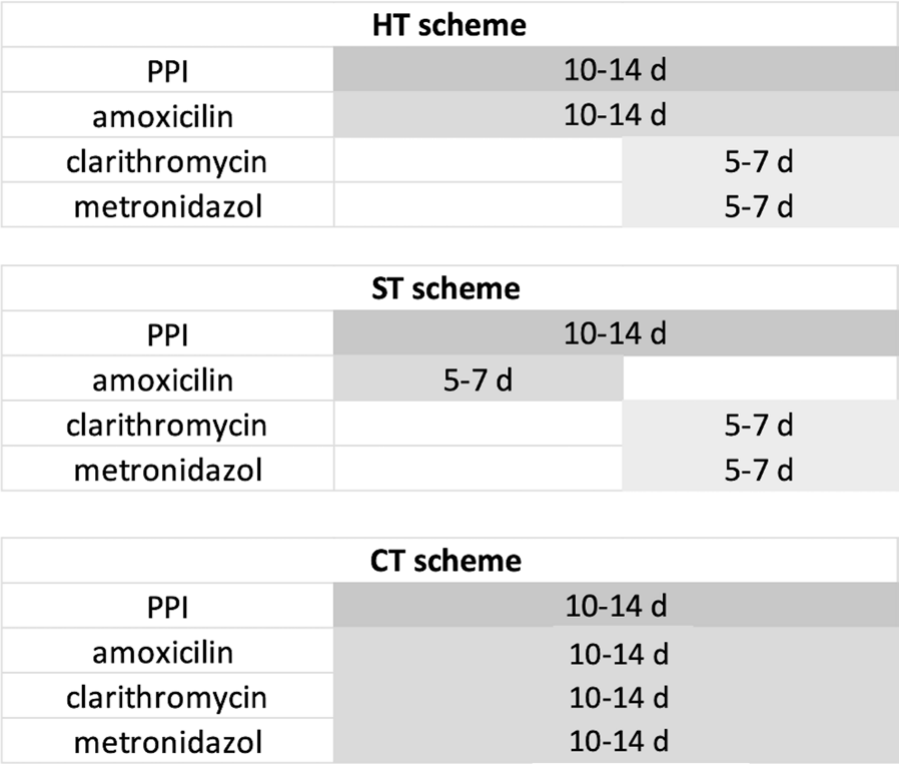
Therapeutic schemes of Hybrid, Sequential and Concomitant regimens, respectively. *PPI* Proton Pump Inhibitor, *HT* Hybrid Therapy, *ST* Sequential Therapy, *CT* Concomitant Therapy

The intervention in this study was HT defined as the administration of any PPI at any dose for 10 to 14 days twice daily; plus, amoxicillin 1000 mg for 10 to 14 days twice daily; plus, the addition of clarithromycin 500 mg or moxifloxacin 400 mg twice daily and metronidazole or tinidazole 500 mg twice daily in the final 5 to 7 days of the treatment (Fig. 1).

HT was compared to other non-bismuth therapies (control groups) used in the treatment of *H. pylori* infection: concomitant therapy (CT) and ST.

CT included patients that took any PPI at any dose, amoxicillin 1000 mg, clarithromycin 500 mg or moxifloxacin 400 mg and metronidazole or tinidazole 500 mg all twice daily for 10 to 14 days. (Fig. 1).

ST, defined as taking any PPI at any dose twice daily for 10 to 14 days, plus amoxicillin 1000 mg twice daily in the 5 to 7 initial days of therapy, followed by clarithromycin 500 mg or moxifloxacin 400 mg and metronidazole or tinidazole 500 mg twice daily in the last 5 to 7 days of therapy. (Fig. 1).

An acceptable eradication rate was defined as equal or higher than 90% in per-protocol analysis, as defended by Graham [25]. Assessment of *H. pylori* eradication was performed 4 to 6 weeks after treatment, using either urea breath test (UBT), histologic assessment (HA) by biopsy with or without rapid urease test (RUT), or stool antigen test (SAT).

Additional outcomes of this review were the comparison of compliance rates and adverse events between the intervention and control groups. Compliance with therapies was assessed either by personal or telephone interview with the patient or by counting the remaining pills after the end of the treatment.

### Information sources and search strategy

The literature was searched electronically on the Cochrane Central Register of Controlled trials library, PubMed, Embase, Scopus, LILACS, and ClinicalTrials.gov. The search term ((helicobacter pylori) AND (hybrid therapy)) was used across all platforms.

**Fig. 2:**
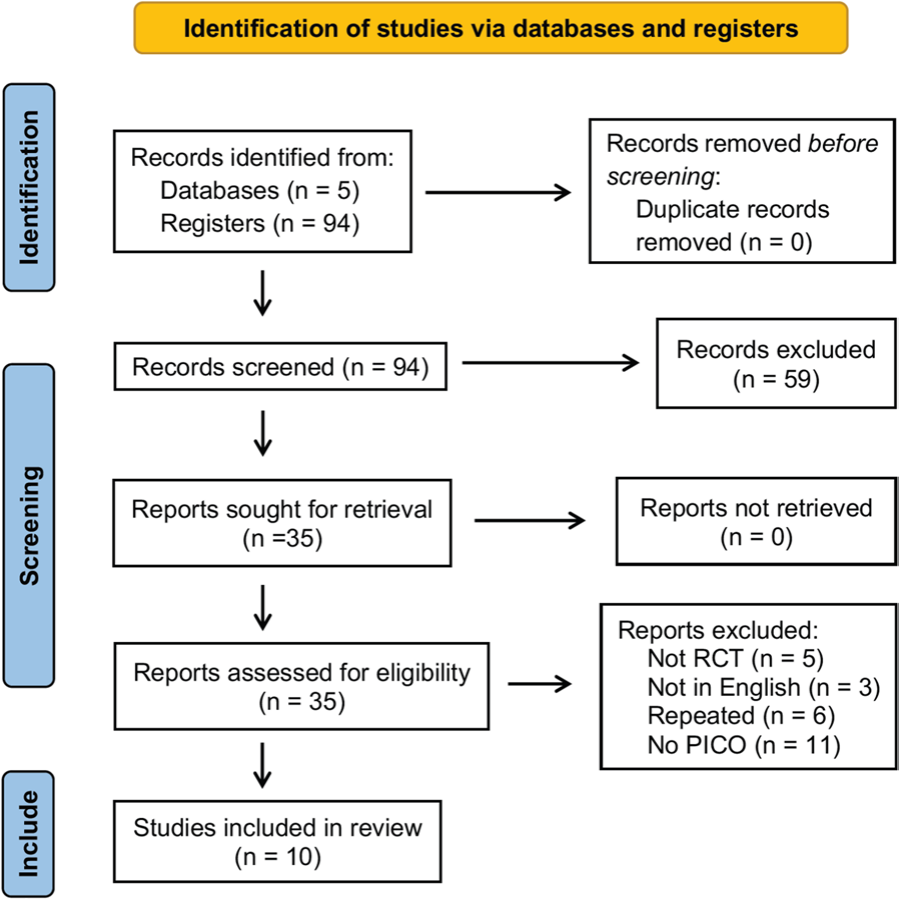
Study selection flowchart. *RCT* Randomized clinical trial, *PICO* Population; Intervention; Comparison; Outcome

Only randomized controlled trials were included. Only articles written in the English language were included. The latest update on the search was performed on May 7, 2021. All studies published before this date were included. The detailed search strategy is illustrated in Fig. 2. References of the studies reviewed were also searched to avoid any exclusion.

### Study selection

The study selection was comprised of two screenings, performed independently by two reviewers (DJM and MJT). Reviewers assessed the abstracts and the titles of the articles. Articles that were deemed highly unlikely to be relevant to the study were excluded. Next, the two reviewers assessed the full-text articles, screening for inclusion criteria according to our defined PICO.

Studies in children, reviews and meta-analysis, reports, letters, editorials, basic research, studies in animals and abstracts with insufficient information were excluded.

The study appraisal was conducted using the Critical Appraisal Skills Programme—Randomised Controlled Trials (CASP-RCT) checklist [26].

### Data collection process and data items

The data extracted included: study design; length of follow-up; patients’ demographics; patients’ symptoms (when applicable); diagnostic methods; number of enrolled participants in the study; number of participants in each group; therapies used in the different groups and their respective dosages; eradication rates (ITT and PP analysis); adverse events and compliance rates for all groups.

### Risk of bias assessment

**Fig. 3:**
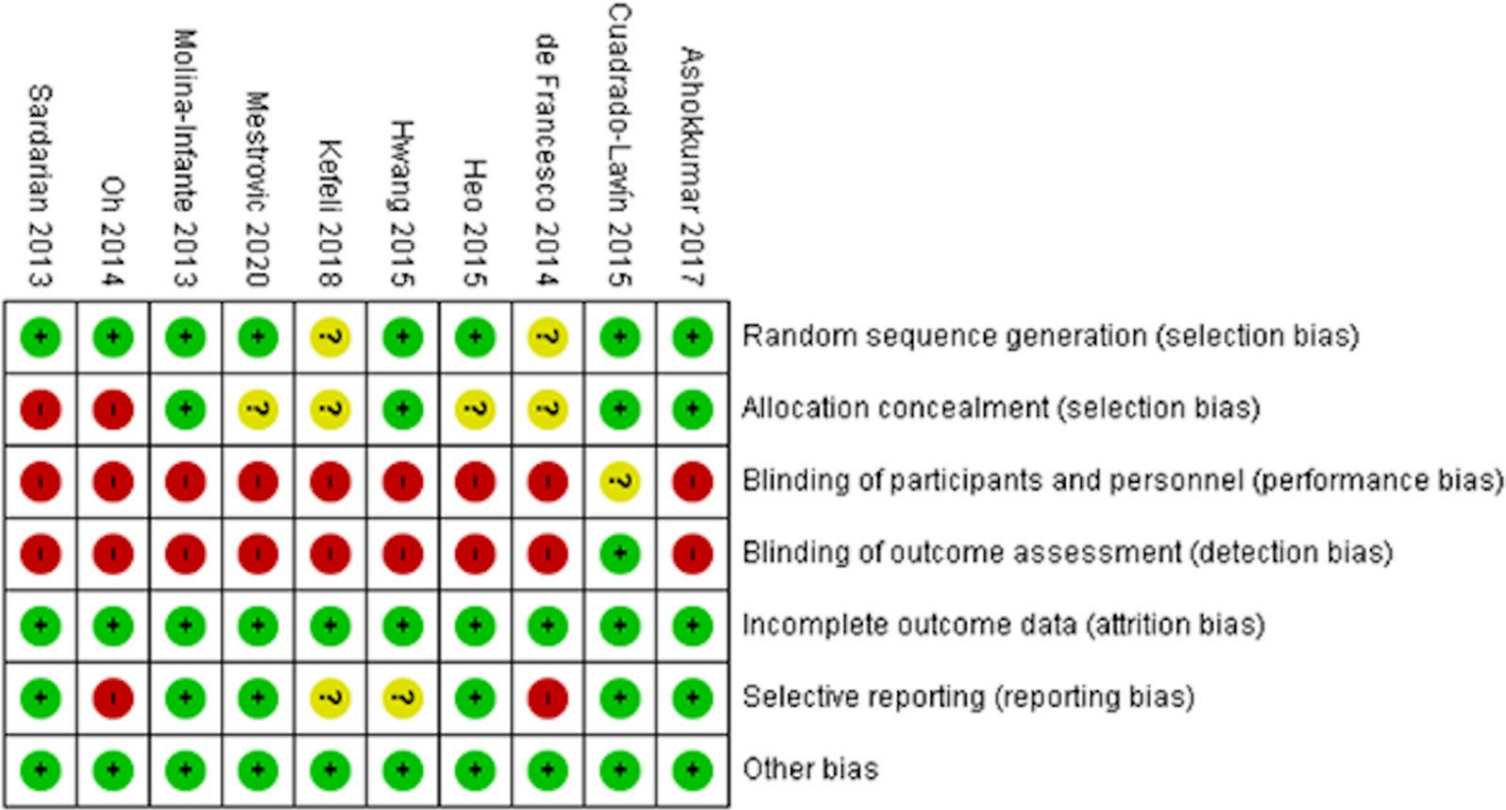
Risk of bias summary: review authors' judgements about each risk of bias item for each included study

Risk of bias of the included articles was assessed independently by two reviewers (DJM and MJT), using the Review Manager (RevMan) version 5.4. The Cochrane Collaboration, 2020. In case of any discrepancies, the reviewers discussed until a consensus was reached. The risk of bias assessment is summarized in Fig. 3.

### Statistical analysis

Statistical analysis was performed using Review manager 5.4 from the Cochrane Collaboration, computing meta-analysis of the studies for the endpoints defined (eradication rates, adverse events, and compliance rates).

The measure of effect considered was the risk ratio (RR) comparing HT versus CT and HT versus ST, and its 95% CI were estimated by the Mantel–Haenszel method using a random effects model. The statistical significance of the overall effect was assessed by the Z-statistic approximation and its p-value interpreted at a 5% significance level. Heterogeneity between studies was evaluated by the Thompson and Higgins statistics and quantified using the I^2^ statistics.

## Results

### Study selection and characteristics

The research that was conducted resulted in 94 entries across the five databases. All 94 records were first screened by two authors (DJM and MJT), who assessed their titles and abstracts. Fifty-nine studies were excluded either because they included a modified regimen of HT (reverse hybrid therapy); they did not compare hybrid therapy to sequential or concomitant therapies; abstracts were not available in the English language. The remaining 35 full texts were retrieved and assessed for our defined eligibility criteria. Finally, a total of 10 studies that met our eligibility criteria were identified. The study selection process is described in accordance with the PRISMA methodology and is illustrated in Fig. 2. A summary of the general characteristics of the included studies is shown in Table 1.

**Table 1:** Summary of the included studies

Publication Year	First Author	Country	HT	Controls	Total Enrolled	Patient Characteristics	Infection diagnosis	Resistance rates	Treatment duration (days)	Eradication	Eradication rates (ITT)%
2020	Antonio Mestrovic	Croatia	71	69 (CT)	140	Dyspeptic symptoms	SAT, RUT, UBT, HA	CAM > 20%, MET 10.2%	14	SAT	83.1
2015	Jun Heo	Korea	241	209 (CT)	422	Dyspeptic symptoms	RUT, UBT or HA	CAM ≥ 20%, MET ≥ 30%	10	UBT	78.8
2015	Jae Jin Hwang	Korea	144	140 (ST)	284	Gastritis and/or Peptic Ulcer Disease	UBT, HA, RUT	CAM 37.3%, MET 35.8%	14	UBT	79.2
2014	Vincenzo De Francesco	Italy	110	330 (ST or CT)	440	Dyspeptic symptoms	RUT and HA	N/A	10 (SQ), 14 (HT, CT)	UBT	82.7
2013	Hossein Sardarian	Iran	210	210 (ST)	420	Gastric or Duodenal Erosions	HA and/or RUT	CAM 30%, MET 73.4%, AMOX 6.3%, DR* 21.2%	14 (HT), 10 (ST)	UBT	89.5
2014	Dong Hyun Oh	Korea	90	94 (ST)	184	Dyspeptic symptoms	RUT or HA	CAM 23.7% AMOX 14.9%	14	UBT	81.1
2013	Javier Molina-Infante	Italy + Spain	171	172 (CT)	343	Dyspeptic symptoms	UBT, RUT, HA or Ct	CAM 23.5%, MET 33%, DR 8.8%**	14	UBT	92
2015	Antonio Cuadrado-Lavín	Spain	120	120 (CT)	300	Dyspeptic symptoms	UBT, HA or RUT	CAM ~ 14%	10	UBT	90.8
2018	Ayşe Kefeli	Turkey	170	170 (ST)	340	Gastritis	HA	CAM 40.2%, MET 45.5%	14	UBT	86.5
2017	Sahoo Ashokkumar	South India	60	60 (ST)	120	Gastritis and/or Peptic Ulcer Disease	HA	CAM 33%, MET 78%	14 (HT), 10 (ST)	HA or RUT	88.3

*RCT* randomized clinical trial, *HT* Hybrid therapy, *CT* Concomitant therapy, *SQ* Sequential therapy, *SAT* stool antigen test, *RUT* rapid urease test, *UBT* urea breath test, *HA* histological assessment, *Ct* culture, *CAM* Clarithromycin, *MET* Metronidazol, *AMOX* Amoxicillin, *DR* double resistance, *ITT* Intention-to-treat

**prevalence of resistance rates in the settings where the trial was conducted

*(clarithromycin + metronidazole)

### Overall eradication rates, adverse events, and compliance rates

The mean eradication rates achieved by HT in the ITT and PP analyses were, respectively, 86% (range: 79.2–90.8%) and 91.7% (range: 82.6–96.1%). Adverse events were 30.7% (range: 12.8–67.5%), 26% (range: 11.8–43%) and 38.9% (range: 14.05–65.8%) in HT, ST, and CT groups respectively. Regarding compliance rates, HT showed an average of 95.7% (range: 87.3–100%); ST had an average of 97% (range: 95–100%) and CT had an average of 93% (range: 87–98%).

### Hybrid therapy versus concomitant therapy

**Fig. 4:**
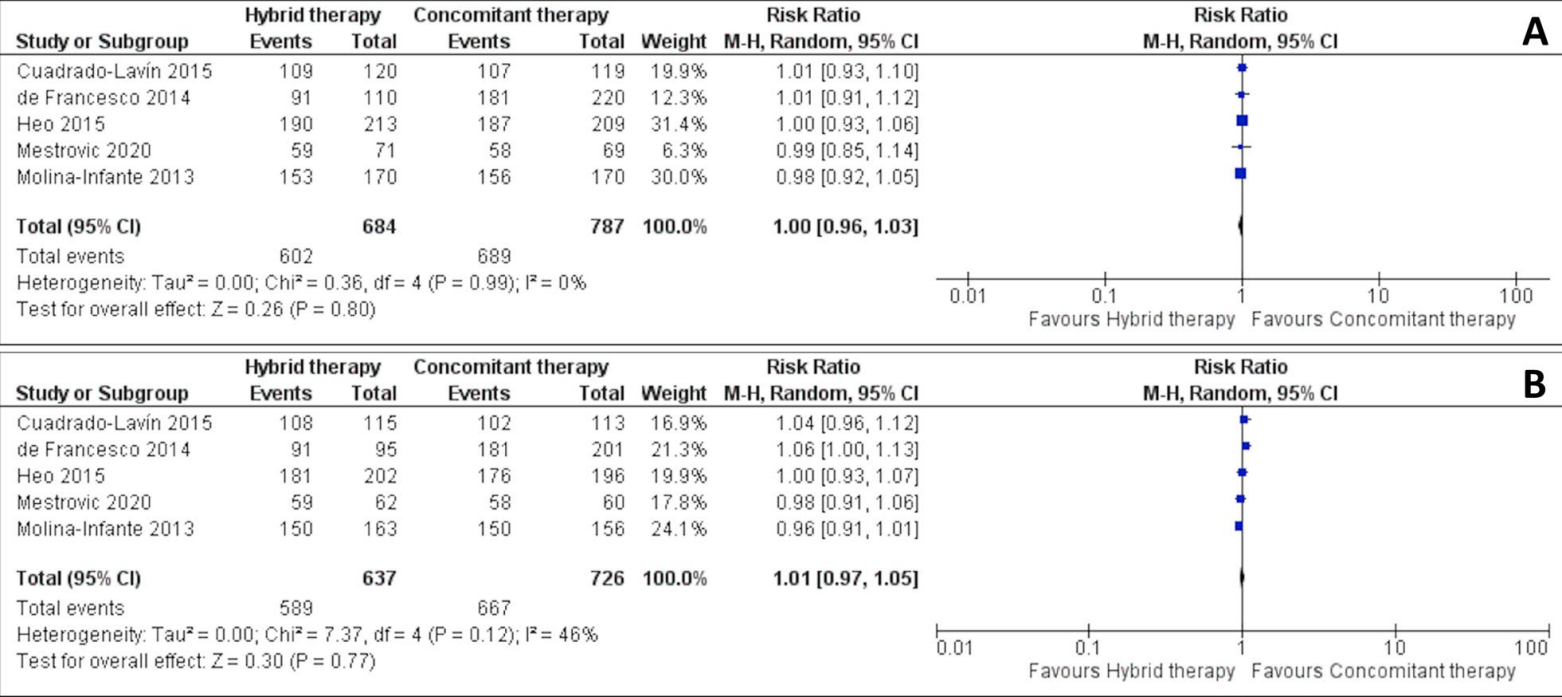
Forest plot comparing eradication rates (intention-to-treat analysis **A** and per-protocol analysis **B**), between Hybrid therapy and Concomitant therapy in the treatment of *Helicobacter pylori*. *M-H* Mantel Haenszel Test, *CI* Confidence interval

HT and CT were compared across 5 studies [21, 27–30], including a total of 1471 patients. According to ITT analysis, the differences in eradication rates between these groups were not statistically significant (RR 1 [0.96, 1.03], p = 0.80, I^2^ = 0%) (Fig. 4A). PP analysis showed similar results, demonstrating that eradication rates did not statistically differ between HT and CT (RR 1.01 [0.97, 1.05], p = 0.70, I^2^ = 46%) (Fig. 4B).

Regarding adverse events, the meta-analysis did not show a statistically significant difference between HT and CT (RR 0.91 [0.80, 1.04], p = 0.16, I^2^ = 5%) (Additional file 1).

The difference in compliance rates between the groups was statistically significant, favouring HT and indicating a higher chance of compliance of 3%, compared with CT (RR 1.03 [1.0, 1.05], p = 0.04, I^2^ = 0%) (Additional file 1).

### Hybrid therapy versus sequential therapy

**Fig. 5:**
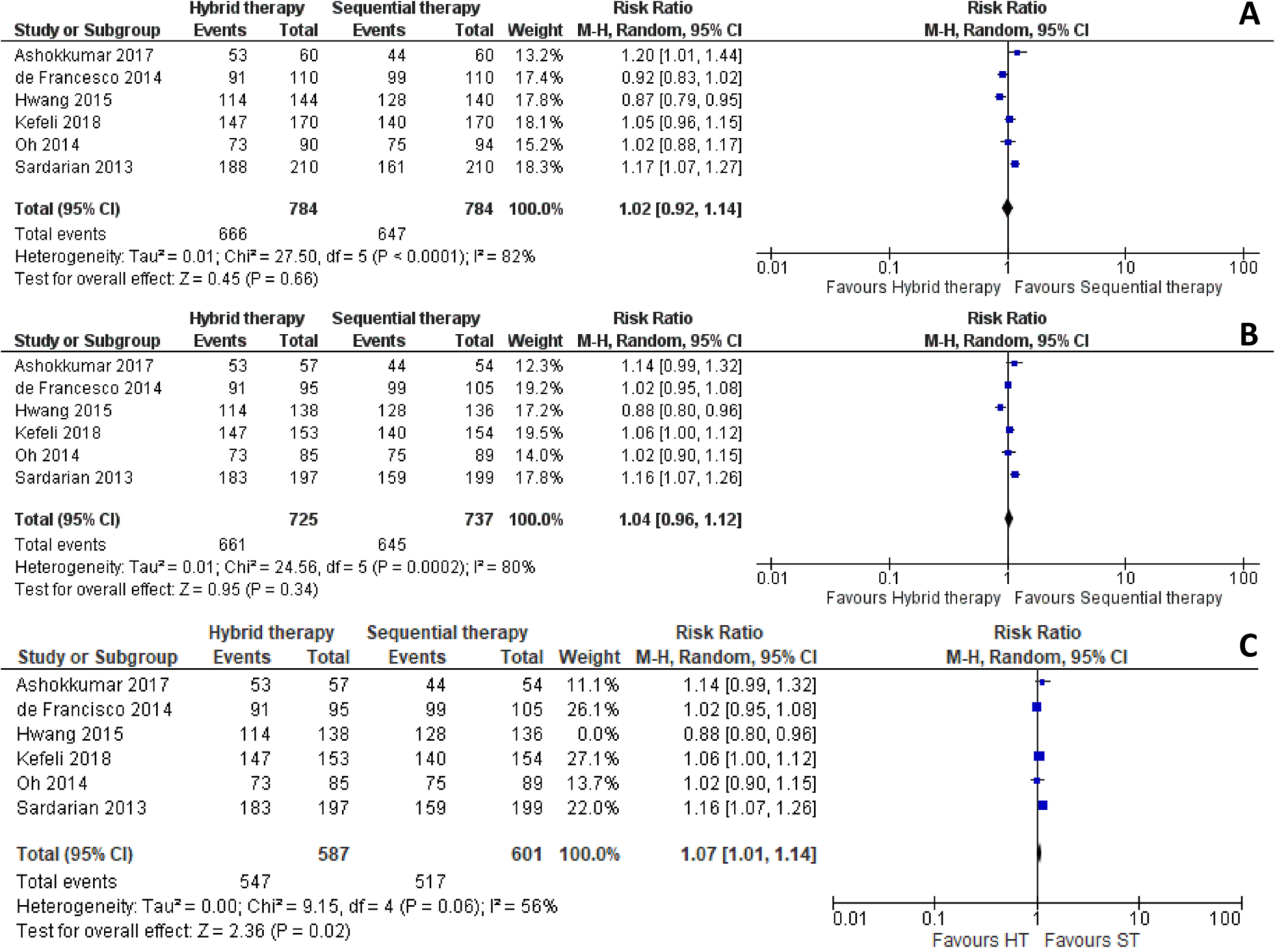
Forest plot comparing eradication rates (intention-to-treat analysis **A** and per-protocol analysis **B** (excluding study with Moxifloxacin **C**)), between Hybrid therapy and Sequential therapy in the treatment of *Helicobacter pylori*. *M-H* Mantel Haenszel Test, *CI* Confidence interval

HT and ST were compared across 6 studies [21, 22, 31–34], reporting on a total of 1568 patients. When it comes to eradication rates according to ITT, the meta-analysis showed significant heterogeneity between the groups, but no statistically significant difference in this outcome (RR 1.02 [0.92, 1.14], p = 0.66, I^2^ = 82%) (Fig. 5A). When analysing PP, the results were similar, demonstrating a high heterogeneity between the groups and no statistically significant difference between HT and ST (1.04 [0.96, 1.12], p = 0.34, I^2^ = 80%) (Fig. 5B). When a sub-analysis, excluding the study with Moxifloxacin of Hwang et al*.*, was performed the only statistically significant difference found was the PP analysis between HT and ST (1.07 [1.01, 1.14], p = 0.02, I^2^ = 56%) that favoured the latter (Fig. 5C).

As far as adverse events are concerned, HT revealed a tendency to an increase in adverse events in comparison to ST, but no statistically significant difference was found (RR 1.10 [0.89, 1.36], p = 0.39, I^2^ = 35%) (Additional file 1).

The compliance rates were not statistically different between the groups (RR 1 [0.98, 1.01], p = 0.46, I^2^ = 0%) (Additional file 1).

## Discussion

HT is a non-bismuth quadruple therapy. This regimen was first proposed and reported by Hsu et al*.* and demonstrated excellent eradication rates, as well as a relevant compliance and safety profile [9]. In subsequent years, this alternative therapy has been recommended as first-line therapy in populations naïve to macrolide including therapies and high *H. pylori* resistance rates to either clarithromycin or metronidazole [35]. This may be particularly relevant in the central region of Portugal, where resistance rates to clarithromycin or metronidazole are worrisome [16]. Another advantage of this treatment regimen over CT is the shorter duration of exposure to metronidazole and clarithromycin, theoretically leading to a reduction in side effects of these antibiotics.

This systematic review and meta-analysis included 10 studies that compared HT with either ST or CT in a population of 2993 patients. To the best of our knowledge, this is one of the largest populations in whom efficacy of HT was assessed with a meta-analysis to this date.

In our study, HT demonstrated similar eradication rates compared to sequential and concomitant therapies.

Among the included studies, HT achieved on average an eradication rate of 86%, which is superior to the recommended eradication rate of 80% in ITT analysis proposed by the Maastricht I Consensus report [36]. Moreover, in this meta-analysis HT achieved an efficacy higher than 90% in the PP analysis as defended to be adequate by Graham et al. [25]. These findings reinforce the power of this regimen to eradicate *H. pylori* proposed by previous studies [7]. In fact, there is still a margin for improvement in *H. pylori* eradication therapies and the role of microbiota in this process should be thoroughly investigated.

Of the 10 included studies, two of them showed that HT was superior to ST [21, 32]; six studies showed similar eradication rates when comparing hybrid to concomitant and/or sequential groups in high antibiotic resistant regions [22, 33]; only two studies demonstrated that HT was inferior to ST [31, 34]. These discrepancies can be partly explained by different antibiotic resistance patterns in those countries and by different compliance rates between the two regimens. Moreover, ST and HT had comparable eradication rates in the PP analysis, but not in the ITT analysis. It is highly likely that patients’ compliance to therapies may had had a role in this discrepancy. Even though adherence is difficult to control, awareness must be reinforced.

In a sub-analysis including only the studies with clarithromycin (excluding the work with Moxifloxacin) a statistically significant difference in the eradication rates according to the PP analysis between HT and ST was found. We attribute this to lesser heterogeneity when excluding this work. Nevertheless, the results from the ITT analysis were similar when the study with Moxifloxacin was included showing similar efficacy of HT and ST.

When comparing adverse events between HT and CT, the analysis revealed that the two groups were not statistically significant, but it demonstrated a tendency for a lower occurrence of adverse events in the HT by 9%. HT and ST did not show statistically significant differences in the adverse events outcome. Regarding compliance rates, HT demonstrated higher tendency for lower adherence to this regimen compared to ST, but it was not statistically significant. In the same groups, there was no difference in the compliance rates, that were considerably high. This can be explained by the inclusion in a trial, that is always a motivating factor for both patients and doctors [37]. This should be taken into consideration in our daily clinical practice. Empathy and communication are crucial to obtain success in *H. pylori* eradication therapies.

The evidence presented in this review is conflicting with the results outlined in a previous review comparing HT with other non-bismuth therapies [19]. Hsu et al*.* demonstrated that HT was more effective than ST, but similar in efficacy when compared to CT. The authors attribute these results to the differences in antibiotic resistance in the populations studied, as well as to the high heterogeneity among individual characteristics of the patients included [9]. In fact, future challenges regarding this matter include the assessment of differences in eradication rates having regional antibiotic resistance patterns in consideration.

Nevertheless, we acknowledge some limitations of this review. Although initially contemplated in the design of the study, a meta-analysis comparing hybrid therapy to standard triple therapy was not feasible, because only one trial with standard triple therapy completed the criteria to be included in our final pool of studies [27]. We consider that this comparison would be relevant to reinforce the loss of efficacy of standard triple therapy. However, triple therapy has already shown a decrease in eradication rates to unacceptable levels [38]. As a matter of fact, a recent report showed a lesser tendency in triple therapy prescription in many European countries [39]. Moreover, antibiotic resistance and its effects on eradication rates were not compared because only one of the included studies reported this outcome [30]. In fact, the majority of studies included patients from the Mediterranean countries, but studies analysing India’s and South Korea’s realities were also included. This may have led to regional differences in antimicrobial resistance rates which may have determined some differences in final results. Another possible limitation of the review is the fact that the included RCTs were not blinded to the treatment regimens attributed to the groups, placing the studies at high risk for performance and detection biases (Fig. 2). We attribute the lack of blinding of the studies to the complexity of the regimens being administered. Therefore, we do not believe that these biases compromise the quality of the included RCTs.

Additionally, the most recent Maastricht VI guidelines defend that it would be reasonable to perform *H. pylori* eradication guided by susceptibility tests (molecular or after culture). However, even the authors accept that the generalised use of such a susceptibility‐guided strategy in routine clinical practice remains to be established. So, empiric therapies are and will continue to be largely implemented in different countries and further investigation about the most efficient and safest treatment methods continue to be essential.

Another problem with quadruple non-bismuth therapies could be dual resistance to both clarithromycin and metronidazole [40]. Theoretically such therapies would have inadequate eradication rates if dual resistance is higher than 15% [40]. Eradication of Helicobacter pylori infection with non-bismuth quadruple concomitant therapy. In: Rahman AU, Choudhary MI, eds. Frontiers in anti-infective drug discovery Bentham science publishers, 2020: 1–34.). However, the present study demonstrates a high success rate of HT even in ITT analysis. So, according to available data, it should be considered a valid option in *H. pylori* treatment.

Our meta-analysis has some key strengths, such as the inclusion of only randomized controlled trials and the low heterogeneity between studies, which powers our statistical analysis. In fact, to the best of our knowledge, this meta-analysis is the most recent, complete, and accurate, with a good level of evidence, comparing HT and other commonly used quadruple regimens. Our work represents a step further in the comprehension of the efficacy of HT in the treatment of *H. pylori*.

## Conclusions

In conclusion, this work demonstrates that hybrid therapy has similar eradication rates to sequential and concomitant regimens. Hybrid therapy also showed significantly less adverse events when compared to concomitant therapy and no significant difference when compared to sequential therapy. Moreover, this study revealed that hybrid therapy had a slightly higher compliance rates when compared to concomitant rates.

In conclusion, hybrid therapy is a favourable option, similarly to sequential therapy, as first-line eradication of *Helicobacter pylori* which may have encouraging clinical benefits in countries with high antibiotic resistance rates.

## Supplementary Information

**Additional file 1.** Forest plot comparing adverse events **A** (fixed forheterogeneity **B**) and compliance rates **C** between Hybrid therapy and Concomitant therapy and comparing adverse events **D** and compliance rates **E** between Hybrid therapy and Sequential therapy in the treatment of *Helicobacter pylori*. *M-H* Mantel Haenszel Test, *CI* Confidence interval

## Data Availability

Not applicable.

## Abbreviations

CASP-RCT: Critical Appraisal Skills Programme–Randomized Clinical Trials
CI: Confidence interval
CT: Concomitant therapy
Ct: Culture
DJM: Dara Jorge Mbanze
HA: Histological assessment
Hp: *Helicobacter pylori*
HT: Hybrid therapy
ITT: Intention-to-treat
LILACS: Latin American and Caribbean Centre on Health Sciences Information
MALT: Mucosa-associated lymphoid tissue
MJT: Maria José Temido
PICO: Population; Intervention; Comparison; Outcome
PP: Per-protocol
PPI: Proton pump inhibitor
PRISMA: Preferred Reporting Items for Systematic Reviews and Meta-analyses
PROSPERO: International Prospective Register of Systematic Reviews
RCT: Randomized clinical trial
RR: Risk ratio
RUT: Rapid urease test
SAT: Stool antigen test
ST: Sequential therapy
STT: Standard triple therapy
UBT: Urea breath test
