# Supplementary figures and images for: Is hybrid therapy more efficient in the eradication of Helicobacter pylori infection? A systematic review and meta-analysis
Source: Ann Clin Microbiol Antimicrob. 2023 Jul 4;22:54. doi: 10.1186/s12941-023-00582-2 (PMC10321015; doi:10.1186/s12941-023-00582-2)

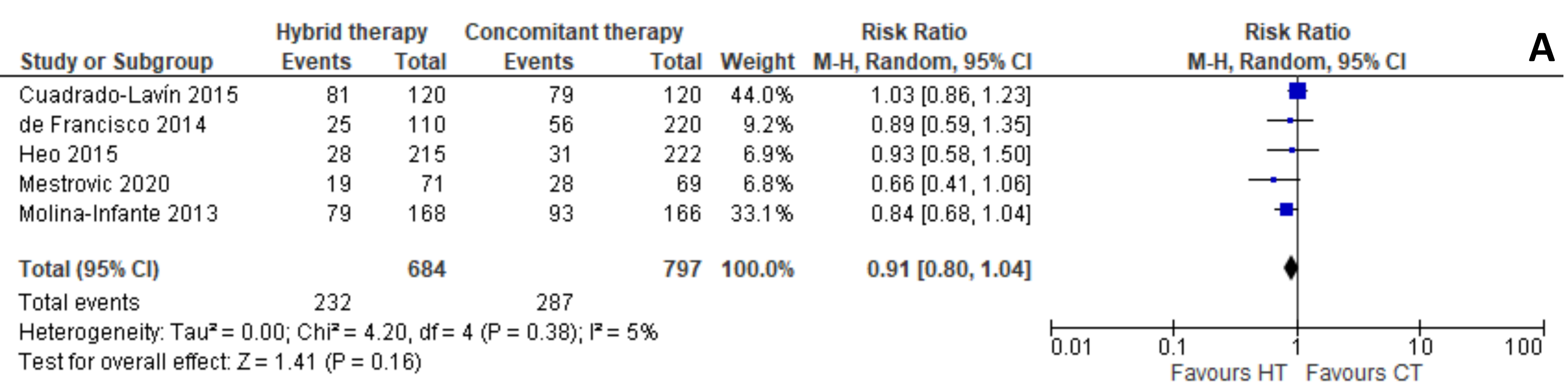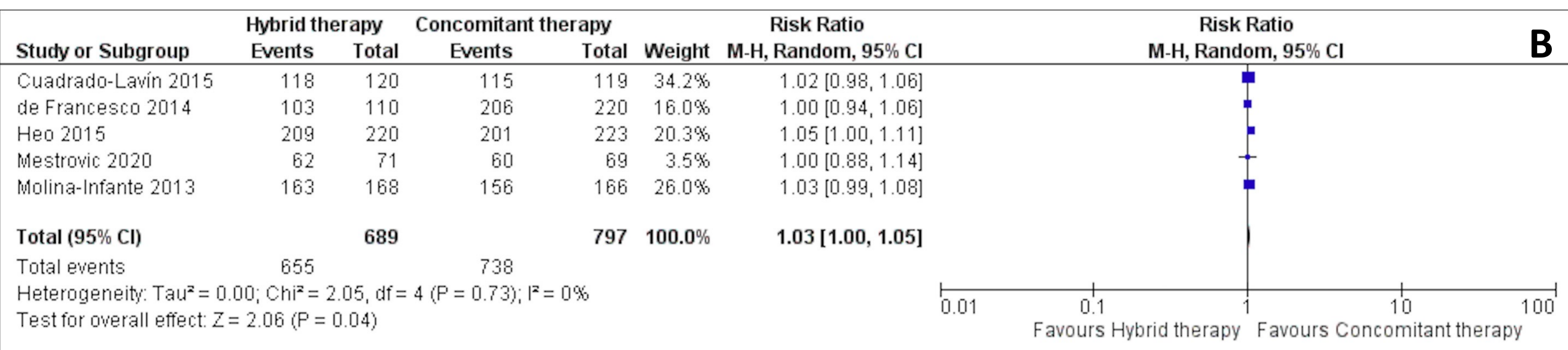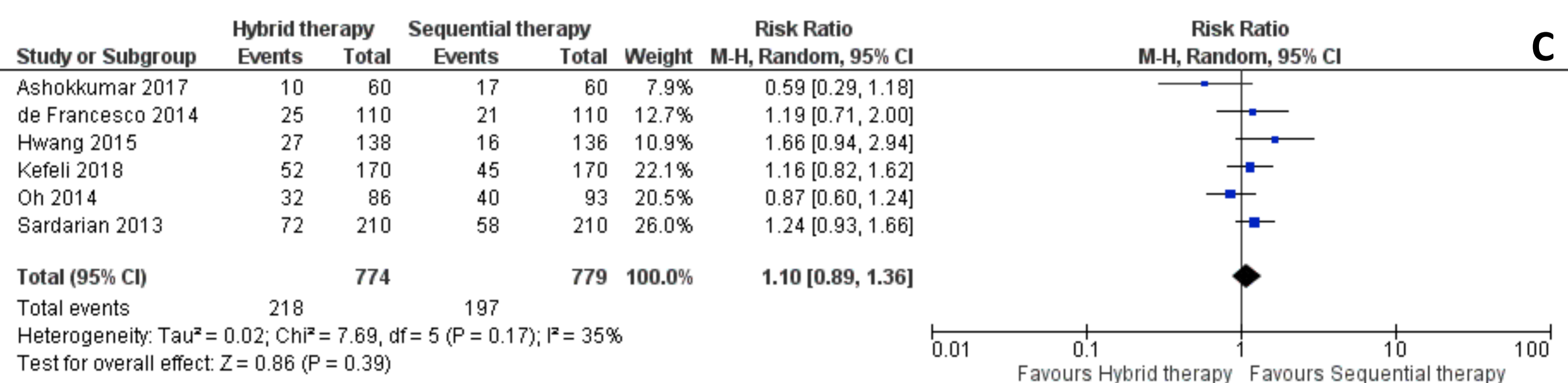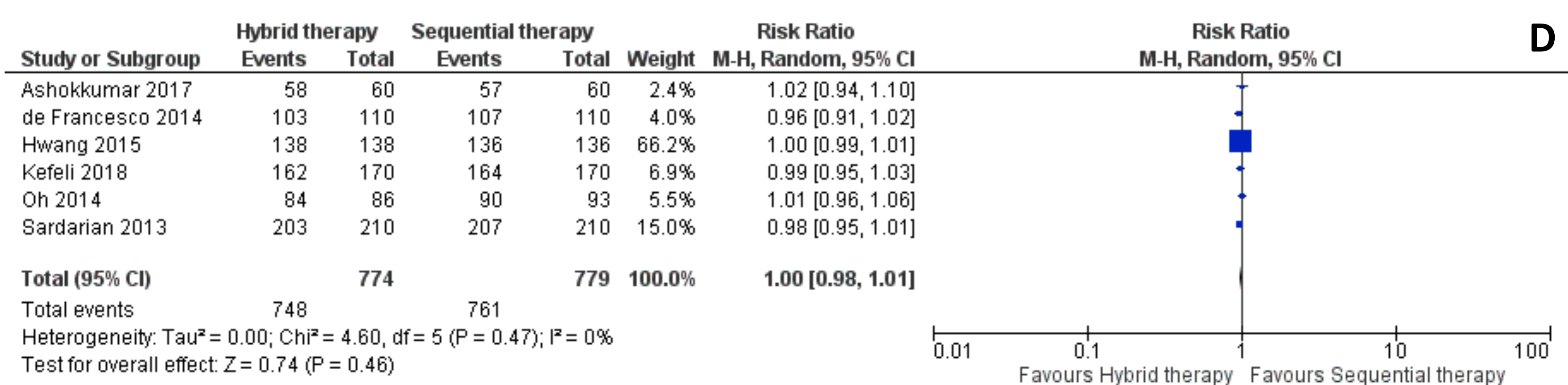

Supplement: Supplementary file 1 — Additional file 1. Forest plot comparing adverse events A (fixed forheterogeneity B) and compliance rates C between Hybrid therapy and Concomitant therapy and comparing adverse events D and compliance rates E between Hybrid therapy and Sequential therapy in the treatment of Helicobacter pylori. M-H Mantel Haenszel Test, CI Confidence interval [file 12941_2023_582_MOESM1_ESM.pdf]
